# Introduction to the Special Issue on Gender and Geoethics in the Geosciences

**DOI:** 10.3390/ijerph13040398

**Published:** 2016-04-01

**Authors:** Mary Thornbush

**Affiliations:** Department of Geography, Brock University, Niagara Region, 1812 Sir Isaac Way, St. Catharines, ON L2S 3A1, Canada; mthornbush@brocku.ca; Tel.: +1-905-688-5500

**Keywords:** sex-based roles, gender equality, feminist approach, academic disciplines, women in science

## Abstract

In this introduction to the Special Issue on Gender and Geoethics in the Geosciences is a focus on the participation of women in traditionally male-dominated professions, with geography as an exemplary academic subject. The Special Issue stems from the Commission of Gender and Geoethics as part of the International Association of Geoethics, and endeavors to bring together efforts at various spatial scales that examine the position of women in science and engineering in particular, as conveyed in engineering geology, disaster management sciences, and climate change adaptation studies. It has been discovered, for instance, that men are more active and personally prepared at the community level (in Atlantic Canada coastal communities), and more action is still required in developing countries especially to promote gender equality and empower women. Studies contained in this Special Issue also reveal that tutoring and mentoring by other women can promote further involvement in non-traditional professions, such as professional engineering geology, where women are preferring more traditional (less applied) approaches that may circumscribe their ability to find suitable employment after graduation. Moreover, the hiring policy needs to change in many countries, such as Canada, where there are fewer women at entry-level and senior ranks within geography, especially in physical geography as the scientific part of the discipline. The exclusion of women in traditionally male-dominated spheres needs to be addressed and rectified for the ascent of women to occur in scientific geography and in other geosciences as well as science and engineering at large.

## 1. Introduction

This Special Issue was originally instigated when the author was asked to coordinate a newly formed Commission on Gender and Geoethics (CoGG) as part of the International Association of Geoethics, (IAGETH), affiliated with the IUGS and IUGG, where she was the representative of the UK national chapter. It was thought that a Special Issue would serve to bring together academics interested in pursuing geoethical concerns within the geosciences. There was much initial interest expressed, as shown on the website of this Special Issue as part of planned papers; however, out of seven, only four planned papers were finally published in this Special Issue addressing Gender and Geoethics in the Geosciences. In the process, not only women expressed interest in becoming a part of this effort, and some European men, for instance, also engaged in feminist research submitted their work for consideration. In fact, the person responsible for the formation of the CoGG is male.

In order to reach an equitable position in society (on a par with men), women need to work with men and other women. This may be an obvious statement, but it is not always practiced. Too often discriminated groups tend to overcompensate when they gain social status or empowerment and dominate in the way that the previous group subverted them (or worse even). To be truly equal, gender harmony is needed, rather than one sex (women or men) dominating the other. Although it may be clear to social scientists, geoscientists, who are in the sciences, may not be familiar with the terminology adopted based on feminist theory. Gender in this Special Issue is based on sex-based roles adopted through socialization. These are socially accepted roles irrespective of sex, so that a gay male, for instance, can partake of a typically female (sex-based gender) role.

The division of labor accepted by our ancestors is sex-specific in Western culture. There is no doubt that women who cross the gender divide of labor are going against societal norms and pose a challenge to the social system. They struggle in various ways. In academia, for instance, women in science and engineering tend to struggle more than women who enter socially accepted disciplines for women; for instance, English literature, social work, nursing, education, psychology, and so on. In the past, women had to fight for access to these disciplines and victory was hard-won after years, so that they are now acceptable academic pursuits for females. Even though more women are earning doctorates, the hiring gap has not closed. Although women’s share of doctoral degrees is rising, the appointment of women to full-time faculty positions has not kept apace, and more women with doctoral degrees are unemployed as full-time faculty. Women with doctorates vary by field of study: lowest in engineering, mathematics, and sciences and highest in education and psychology [1]. Women’s ranks in academic positions are as follows (after [1]): Assistant Professor; Associate Professor; Full Professor (lowest). There is an international trend for women to be less represented at senior ranks, *i.e.*, Full Professor, especially in Japan and least in the US and Canada.

## 2. Women in Traditionally Male-Dominated Academic Disciplines

### 2.1. Women in Geography

In geography and the geosciences, in particular, women have had to struggle for acceptance. Based on a preliminary Internet search of academic faculty websites, the author performed simple (sex-based) counts of listed academics in geography departments at Canadian universities in 2009 (More specifically, the author visited faculty websites of all geography departments (namely, Geography Departments across Canada) listed by the Canadian Association of Geographers [2]. This included several departments, some at the same university but different campuses, and excluded non-universities (colleges) and student societies or clubs. These websites were visited on 1 January 2009 (before the global economic crisis really took hold of university departments) and tallies were obtained based on specific criteria. First, gender was established based on the photographs of faculty webpages or (in the absence of a photograph) on first name, where available. This first step established male or female group membership and is the defining basis of (sex-based) gender in this study. Second, research area of expertise and/or interests were considered to refine whether these were human or physical geographers. Physical geography included traditional subdisciplines: geomorphology; hydrology; climatology; biogeography; and soil science. Further inclusions to this were GIS and environmental scientists, who are typically excluded from traditional conceptions of physical geography. They were included in order to expand the sample size; however, this is contestable. Finally, tallies were established for each department and for all universities across Canada. Consideration was given to rank (whether Assistant, Associate, or Full Professor). Permanent (tenure-track/tenured) and temporary (contracted/limited term appointments) staff were not differentiable using this method.). Overall in geography, 25% more male Assistant Professors were hired; 39% more males were at the Associate Professor level; and 72% more males as Full Professors. This can be divided into subdisciplines (human versus physical geography). In human geography there were 35% more males hired (at all ranks), including:

- 12% more male Assistant Professors;
- 29% more male Associate Professors; and
- 58% more male Professors.

The disparity was found to be greater in physical geography, where 62% more males were hired (at all ranks), including:

- 40% more male Assistant Professors;
- 49% more male Associate Professors; and
- 83% more male Professors.

This information is summarized in Table 1 and Table 2, which compare participation across human versus physical geography, respectively. The purpose of doing this is to test for the effect on academic appointments within the geographical sciences in particular against human geography, which has tended to be more social in scope and traditionally more accepting of women.

The data indicate that fewer women are represented as faculty in physical geography at all levels (96 women in human geography versus 58 in physical geography). Moreover, their numbers (for women) are especially low at the highest rank (of Full Professor) and also at the introductory rank of Assistant Professor; the same trend is evident for women in human geography, so that it is diagnostic of geography overall. So, this suggests that although there are fewer women academics in geography (see Table 3), they appear in yet lower numbers in physical geography.

It has been found that faculty from the humanities and social sciences are more likely (than faculty from science and engineering, for instance) to consider ethical issues, such as those involving gender as well as power, justice, class, and race [4]. This could be limiting the sciences, in particular, where gender equity is an ethical issue that would not commonly be considered.

Since the focus on equity for women in geography published by *The Canadian Geographer* (2002), there has been a time of general disinterest (at least among Canadian publications). American efforts have continued, however, and the American Association of Geographers (AAG) in particular has formed a Committee on the Status of Women in Geography. This committee has recently sought, for instance, women to join (as mentor or mentee) a newly established Mentoring Network for Women in Geography (CSWG) to be launched at its 2016 annual meeting (The author may be contacted by those readers wishing to participate in this initiative). Mentoring is another way that women can become established in geography. Such efforts can help to establish geography as an inclusive academic discipline. This is especially important for physical geography, where it seems that most of the sex-based gender exclusion has occurred and continues to propagate perhaps even now (Admittedly, the current study needs to be repeated in order to verify contemporary counts and trends based on the method deployed here. It would also be interesting to check for any effects since the economic crisis).

#### Discussion

Research has conveyed that women are still in the “A” ranks (namely, Assistant, Associate, and Acting) in academe [5]. It has been postulated that this trend could be due to a mismatch between feminist models and hierarchical systems of academic advancement. After all, gender differences have been found by other researchers in rank and tenure status [6]. Academic feminists have addressed hegemonic control that is embodied specifically in the tenure review process [7] that may have hampered women’s progress to the top of the academic ladder. Needless to say, women are not always victim to just men’s limitations, but may also be affected by other women (such as married women with mothering responsibilities), particularly if they are single academics without children [8]. After all, women have the added burden of bearing children and caring for them that accompanies their sex. These responsibilities may deter them in their climb up the academic ladder, as they progress from the initial stages (as Lecturer and Assistant) to the upper ranks (of Associate and Full Professor), making it more challenging for them to progress in their careers.

Berg and Longhurst [9] have already addressed masculinity in geography at large from an Anglo-American perspective. It has been argued that such “masculinities views” are especially apparent in performing fieldwork [10], where women have been excluded and discouraged from excelling at various levels of their careers (from undergraduate to senior academic levels). This has constituted a form of gender discrimination that has especially affected women in physical geography, and this author argues that it is prevalent in geomorphology more specifically within physical geography. Future studies should actually compare women’s experiences in the geosciences at large, including physical geography as well as geomorphology and other geological or Earth sciences.

These problems transcend location, and affect women more broadly than Canada. For instance, research in the UK investigated the participation of women in academia within physical geography [11]. Through the administration of a survey to women in physical geography at the doctoral level and beyond, they discovered that women were underrepresented (in 1998) and mainly young women (less than 40 years of age) occupied the lower ranks of academia, including at the entry level (as Lecturers). This indicates that there was then little progression in British universities for women in physical geography. Other studies have portrayed this problem as far-reaching, affecting The Netherlands, Catalonia, Hungary, and Singapore in addition to Canada, the USA, and the UK [12]. The cross-national approach taken in this latter study enabled further considerations in addition to equal gender-based representation in academic geography, as having curricular implications and impacting the student experience. Graduate women students in geography have examined various issues regarding geographical inquiry, fieldwork, and childcare and concluded that the experience is gendered [13]. These issues have also filtered into the undergraduate level at British universities, for instance, marring the experience of women students, who have experienced gender inequality and discrimination, but have kept quiet about it as a type of “coping mechanism” [14].

The legacy of the past, arguably, continues to be perpetrated today. In the 18th and 19th centuries, middle-class women became unpaid “invisible” assistants (at home) to their fathers and husbands at a time when they were barred from universities [15]. Daughters as well as companions and wives worked for men in an informal employment, not receiving any public credit. In the present day, it seems that this legacy continues, with women gaining access to academic positions at universities through kinship (nepotism or “cronyism” *cf.* [16]) or as companions of a sort. Other avenues of entry have included religious and ethnic group affiliations. It is a rarity for women to make it as physical geographers even in the modern-day on their own without any association with men or social networks.

Academia is a very political place; it is truly cutthroat [8]. For this reason, it is imperative to open a dialogue (*cf.* [17]) between women in the geosciences in order to share experiences and work towards improved gender parity in such male-dominated disciplines. Specifically within geography, it is advised that more gender content be integrated into traditional courses at the undergraduate level [18]. This would sensitize undergraduates to women’s issues early in their careers and, so, lubricate the way to the acceptance of women within the discipline and especially in physical geography, where women are most underrepresented.

### 2.2. Women in Science and Engineering

The struggle for women continues in traditionally male-dominated disciplines in the sciences and engineering. In engineering and the physical sciences, in particular, women seem to struggle more than in the biological sciences, so there is a range of resistance against the participation of women within these disciplines. In this Special Issue, for instance, engineering geology [19] is considered as exemplary of an area where a gender barrier still exists. In particular, women students have been known to “leak out” of professional engineering geology programs. In their selection of a final year project at the University of Salamanca in Spain, for example, women are selecting more theoretical final projects (that are reflective of traditional geology) over more practical (applied) ones, which could compromise their ability to find work after completion of their degrees. It was discovered [19] that tutoring and mentoring are key aspects of ensuring that more women enter into engineering geology because they are exemplary and provide role models for studies and career orientation.

Also in this Special Issue, Turkish authors [20] examine discipline-specific sex discrimination, as for instance of gender inequality existing in disaster management sciences. A woman-oriented approach is stipulated when addressing gender inequality. International standards are required for assessments, and factors that are relevant to gender should be a part of these standards, as for instance women’s health and hygiene, and should comprise gender-sensitive methods of disaster mitigation and prevention. More broadly, as concerns geoethics in mineral exploration, development, and mining projects [21], efforts of local communities (including women) are necessary in order to check environmental impacts. It is important to also consider gender-based experiences and perceptions, as by [22], around climate change adaptation, as in coastal communities located in Atlantic Canada. This approach has made it possible to discern, for example, that men were more active in these communities and more personally prepared. Moreover, the traditional division of labor is lending to such a gender bias (for action and disaster risk reduction and adaptation) in communities.

## 3. Gender in the Millennium Development Goals (MDGs)

From the local to the global, it is necessary to apply good geoethics at a breadth of scales. Local communities have a limited geographical range and impact, so that national to international efforts are necessary. The MDGs are an example of an international effort to curb poverty (affecting women, but not exclusively) and improve conditions in developing countries. Briefly, the MDG addressing gender (Goal 3) sought to “promote gender equality and empower women.” However, this targeted developing countries and only lower levels of education (primary) achieved this goal mainly due to the discovery that “gender disparities are more prevalent at higher levels of education” ([23], p. 20), with the secondary level being more affected than the primary level, and so forth upwards.Considerable disparities still exist in all developing regions for tertiary education enrollment. This spills over into employment, where “Women’s status in the [labor] market is improving, but gender disparity still exists” ([23], p. 21). The findings also reveal that, “In addition to a lower likelihood of being employed than men, women are far more likely than men to have part-time jobs and be in time-related underemployment” ([23], p. 22), and this is likely to also be reflected in developed countries. Interestingly: “The proportion of women in part-time employment is more than double that of men in almost all countries where data are available. These higher part-time employment rates are associated with a number of factors, including gender inequality in family roles, the absence of adequate and affordable childcare and elderly-care facilities, and/or other social perceptions which play a significant role in the participation of women in employment, in their occupational choices, and in the employment patterns that reinforce gender disparities in the [labor] market.”Finally, even though there is a noted greater participation of women in politics, as conveyed by more seats being held by women in parliament, quotas alone are not enough and more women candidates need to be fielded. However, there has been an inclination for women to hold more “hard” ministerial portfolios, such as in Defense, Foreign Affairs, and the Environment, than their typical “soft” portfolios as part of Social Affairs, Education, and Women’s Affairs [23]. Action is still needed, however, to ensure that gender equality is achieved in all fields (*cf.* [24]). This should cross into their professional careers, rather than just women’s household roles. However, the domestic sphere itself poses a real barrier, as it reflects “very complex domestic institutional structures and cannot be easily and rapidly changed by any amount of outside intervention” ([25], p. 744), and continues to impound on indicators of gender equity.

## 4. Moving Ahead

The competition for jobs continues to shape gender interactions. In the past, as after World War II, when men returned from battle, the “proper” or socially accepted place for women was “in the home” in order to partake of the “nuclear” (traditional) family. It was then possible for women to stay at home and be housewives and stay-at-home moms. These days, the economy (rather than warfare) is driving what is acceptable, and dual income families are necessary in a world that is becoming increasingly expensive, as the cost of living is driven up by resource scarcity amidst a growing world population. So, does this mean that the 1940s housewife is a figment of a bygone time?

This brings to mind an exchange with young women in a mandatory undergraduate course, which had over 200 students enrolled and many of them attended interdisciplinary seminars delivered by a small team of multidisciplinary faculty. I had finished delivering a talk with a male colleague, where I presented the data regarding the under-representation of women among physical geography faculty in Canada. During the question-answer session that followed, one of the students (a young woman) asked why I thought that there were so few women in physical geography. I answered saying that women are being excluded, as conveyed by the numbers. It is possible, after all, to quantify sex-based gender discrimination as a support or proof of its occurrence by undergoing such Internet searches as relayed earlier in this editorial piece. The young woman was not convinced by my response and suggested that perhaps it is in our genetic makeup to behave in the way we do. If this were the case, then I would not be standing in front of them that day, delivering a talk, and instead be at home baking cookies.

We need women to question the norms, to step out of the cookie cutter and challenge what it means to be a woman. Mentoring is one of the most obvious ways of achieving this. But, to do so, we need women mentors—women who are accepted and valuable members of academic departments in the sciences and engineering. Women that cross boundaries and take a stance for what they believe in, to be what they choose to be, rather than blindly following the dictations of society. Sure, for some women, the cookie-cut model continues to fit and suit their needs just fine; but I refuse to believe that this should fit all women. Certainly not in the developed world, and most certainly not in the 21st century.

## 5. Conclusions

The purpose of this Special Issue has been to identify current struggles in gender equality. Its focus has been on gender bias affecting professions. Persistent gender barriers to women in the sciences and engineering in particular are subject here, with papers outlining struggles in professional engineering geology as well as broader geoethical issues at various scales, from the community to international levels. Male domination in households spills into communities (where men are more active and personally prepared) and into national to international politics and governance. Education is a starting point for equality; however, networking and application of expertise as professionals is crucial for the advancement of women’s status and for achieving parity with men. Women are still not earning on-parity with men in most countries, even in the developed world, and they are being excluded from certain professions, such as science and engineering; physical geography has been the focus here, but there are other similarly male-dominated academic disciplines, such as archeology (disciplines where “old boy” networks still prevail). Tutoring and mentoring by women (exemplary leadership) is necessary so that more women enter into traditionally male professions, including geography and the geosciences. A hiring system that is truly equitable, where an equal number of men and women are represented is also much needed for the progression of women’s liberation as equals to men.

## Figures and Tables

**Table 1:** Gender-based representation of faculty in human geography at Canadian university geography departments.

Human Geography:	Female	Male	*Total*
Full Professor	22	82	*104*
Associate Professor	41	75	*116*
Assistant Professor	33	42	*75*
*Total*	*96*	*199*	*295*

**Table 2:** Gender-based representation of faculty in physical geography at Canadian university geography departments.

Physical Geography:	Female	Male	*Total*
Full Professor	11	119	*130*
Associate Professor	28	82	*110*
Assistant Professor	19	44	*63*
*Total*	*58*	*245*	*303*

**Table 3:** Gender-based representation of faculty in geography departments at Canadian universities.

Geography:	Sex-Based Gender	No. of Geography Faculty at Each Rank	% Total Geography Faculty *
Full Professor	Female	33 (13)	5.52 (2.37)
	Male	201 (245)	33.61 (44.63)
Associate Professor	Female	69 (45)	11.54 (8.20)
	Male	157 (157)	26.25 (28.60)
Assistant Professor	Female	52 (31)	8.70 (5.65)
	Male	86 (58)	14.38 (10.56)
*Total*	*Female*	*154 (89)*	25.76 (16.22)
	*Male*	*444 (460)*	74.24 (83.79)

* Percentages from [3], p. 250, Table 4 from 1999 in brackets. It is noteworthy that while Berg [3] based his counts on the Canadian Association of Geographers Directory 2000, the method employed here for data acquisition was based on information derived from faculty webpages.
